# Association between maternal satisfaction with breastfeeding and postpartum depression symptoms

**DOI:** 10.1371/journal.pone.0242333

**Published:** 2020-11-17

**Authors:** Juliana Castro de Avilla, Camila Giugliani, Agnes Meire Branco Leria Bizon, Ana Cláudia Magnus Martins, Andrea Francis Kroll de Senna, Elsa Regina Justo Giugliani

**Affiliations:** 1 Graduate Program in Child and Adolescent Health, Faculty of Medicine, Universidade Federal do Rio Grande do Sul (UFRGS), Porto Alegre, RS, Brazil; 2 Graduate Program in Epidemiology, Faculty of Medicine, Universidade Federal do Rio Grande do Sul (UFRGS), Porto Alegre, RS, Brazil; Curtin University, AUSTRALIA

## Abstract

**Background:**

Due to the multiple health benefits of breastfeeding, it is essential to identify factors that may negatively interfere with this healthy practice. Among such factors are postpartum depression (PPD) and maternal satisfaction with breastfeeding. The objective of this study was to evaluate the association between maternal satisfaction with breastfeeding and symptoms of PPD in the first month after childbirth.

**Methods:**

This cross-sectional study nested in a cohort study was conducted in Porto Alegre, Brazil, with 287 puerperal women selected at two maternity hospitals, one public and one private. Women were interviewed at their homes the week after the infant completed 30 days of life. A structured questionnaire was applied, as well as instruments to evaluate maternal satisfaction with breastfeeding (Maternal Breastfeeding Evaluation Scale) and to screen for PPD (Edinburgh Postnatal Depression Scale). The association between higher satisfaction with breastfeeding (outcome) and negative PPD screening test was assessed using Poisson regression with robust variance, adjusting for specific covariables. Adjusted prevalence ratios (aPR) and respective 95% confidence intervals (95%CI) were estimated.

**Results:**

The prevalence of increased satisfaction with breastfeeding (defined as women with scores above the median) was 47% higher among women who screened negative for PPD when compared to those with a positive result (aPR 1.47; 95%CI 1.01–2.16). This result was adjusted for maternal age and skin color, cohabitation with the infant’s father, planned pregnancy, type of delivery, exclusive breastfeeding, and occurrence of breastfeeding problems.

**Conclusions:**

The findings of this study showed an association between higher maternal satisfaction with breastfeeding and absence of PPD symptoms, reinforcing the importance of caring for the mental health of pregnant and puerperal women and paying attention to their satisfaction with breastfeeding.

## Introduction

Traditionally, breastfeeding has been studied with a focus on the biomedical aspects of lactation, prevalence rates and risk factors associated with breastfeeding initiation and cessation, often neglecting the complex network of sociodemographic, economic, cultural and emotional factors implicated in the process [1, 2]. More specifically, maternal satisfaction with breastfeeding has not been taken into consideration in most studies designed to assess breastfeeding success [3].

From the mothers’ perspective, it has been suggested that satisfaction with breastfeeding is a success factor for this practice [4], and that it is influenced by aspects related to the quality of the experience of each woman, the infant’s ability to nurse, maternal self-confidence, early maternal satisfaction resulting from skin-to-skin contact, stay in rooming-in settings, and encouragement to breastfeed on demand [5–7], not to mention the physical and mental health of the breastfeeding woman [8].

The postpartum period is characterized by major transformations in the woman’s life, with a potential risk for the onset of psychological disorders. One of the most common psychopathologies in this period is postpartum depression (PPD), which may manifest any time from the prenatal period up to several months after delivery [9]. In Brazil, the estimated prevalence of PPD ranges from 7.2 to 39.4%. This great variation is probably due to differences in the evaluation methods and cutoff points adopted, the timing of assessment, as well as cultural and social characteristics of the studied populations, especially considering the significant economic and social disparities observed across Brazilian regions [10–12].

Some studies have associated maternal depression with an increased risk for early interruption of breastfeeding [13–16]. Other studies, in turn, have suggested that breastfeeding is a protective factor against the onset of depressive symptoms in the mother, and that breastfeeding interruption is a stressor involved in the development of the disorder [17, 18]. Therefore, there seems to be a bidirectional association between early weaning and PPD [12, 14, 18, 19] and we speculate that maternal satisfaction with breastfeeding may be involved in that association. Some evidence of the association between maternal satisfaction with breastfeeding and breastfeeding duration is available in the literature [5]. However, up to the present moment, no previous study has been designed to assess the association between satisfaction with breastfeeding and presence of PPD symptoms. The present study was motivated by this gap in the literature.

Therefore, the objective of this study was to assess the association between maternal satisfaction with breastfeeding (outcome) and the presence of PPD symptoms in the first month postpartum.

## Methods

This was a cross-sectional study nested in a cohort study whose population consisted of women who gave birth at two large-scale maternity services in the municipality of Porto Alegre, southern Brazil, one public and one private. The public maternity service is part of a general university hospital accredited by the Baby-Friendly Hospital Initiative. The private maternity service is also located within a general hospital, but is not affiliated with any university. Both are reference maternity hospitals for usual- and high-risk deliveries, and both services practice rooming-in as routine.

Every day, from January to July 2016, two women were selected from the public maternity service and one from the private service. This proportion was adopted to result in a sample that would be similar to the Brazilian population in terms of the use of public/private health services: 60 to 70% of the Brazilian population use solely the public health system [20]. The women were randomly drawn by lot and selected among those eligible for the study. For the draw, two spheres of identical texture and dimensions were used with the words "yes" and "no." They were removed from a dark colored envelope by one of the researchers, preventing any visual or tactile distinction between the spheres. If the selected woman refused to participate in the research, a new drawing was carried out to replace her. The eligibility criteria were: mothers residing in the municipality of Porto Alegre; having given birth to live-born term singletons (gestational age ≥ 37 weeks); having breastfed at least once; absence of neonatal complications and/or malformations that could interfere with breastfeeding; and absence of mother or child problems that resulted in admission of either one to the intensive care unit. To ensure the safety of the research team, women who lived in especially violent areas were not included. In those areas, health authorities have restricted home visits for security reasons.

Following selection by draw, women were invited to participate in the study, and those who agreed to participate were requested to sign an informed consent form. Then, a date was set for an interview, which should take place at the woman’s home or at a location of her choice, the week after the child completed 30 days of life. The interviews lasted for approximately 60 minutes and were conducted by one of a team of 10 interviewers, all from the health field and previously trained for the task. A structured questionnaire specifically designed for this study was used to collect sociodemographic data, information on pregnancy, delivery, and immediate postpartum period, in addition to questions related to the health of both the mother and the child, and child feeding habits, in the first 30 days postpartum. In addition to this questionnaire, the women were asked to complete two self-report instruments: one to assess maternal satisfaction with breastfeeding and another to detect symptoms of PPD.

The outcome “maternal satisfaction with breastfeeding” was measured using the Maternal Breastfeeding Evaluation Scale (MBFES), designed by Leff et al. [21] and validated for use in Brazil [22]. The original instrument comprises 30 questions and includes three subscales: pleasure and fulfillment of the maternal role; child satisfaction and growth; and maternal lifestyle and body image. Each item has 5 Likert-type answers; the total score may range from 30 to 150 (higher scores indicate greater satisfaction). The Brazilian validation process resulted in the exclusion of one item, which elicited feelings that were perceived differently in the original instrument and in the Brazilian population [17]. Therefore, the instrument used in the present study comprised 29 questions, with a possible total score varying from 29 to 145.

Finally, women were screened for PPD using the Edinburgh Postnatal Depression Scale (EPDS), validated for use in Brazil by Santos et al. [23]. A score ≥ 11 indicates a positive screening for PPD, as recommended in the Brazilian validation study [23].

To ensure data collection quality, one of the investigators checked part of the responses given by 5% of the sample, randomly selected, via telephone contact.

Sample size was calculated using the Epi-Info software. A minimum sample size of 252 women was estimated using the following parameters: confidence level of 5%, power of 80%, prevalence of greater satisfaction of 50%, prevalence of negative screening test results for PPD (score < 11) of 85% [23], and prevalence ratio of 1.5.

Statistical analyses were performed using the Statistical Package for the Social Sciences (SPSS) version 21.0 (IBM Corp., Armonk, USA). Initially, a descriptive analysis was performed using the measures considered adequate for each variable (i.e., mean and standard deviation for variables with normal distribution and median and interquartile range for other variables) and the respective 95% confidence intervals (95%CI). Then, a univariate analysis was performed using Pearson’s chi-square or Fisher’s exact test for categorical variables, and Student’s *t* or Mann-Whitney test for continuous variables. For the multivariate analysis, the following covariables were tested because of their known association with breastfeeding [24–26]: maternal age (continuous variable), maternal skin color (white vs. non-white), socioeconomic level (A/B vs. C/D/E, with levels A and B indicating higher socioeconomic strata [27]), maternal schooling (primary school = 8 years vs. secondary and higher education), parity (primiparous vs. multiparous), cohabitation with partner (yes vs. no), planned pregnancy (yes vs. no), newborn sex (male vs. female), newborn birth weight (continuous variable), type of delivery (vaginal vs. cesarean), type of maternity (public vs. private), exclusive breastfeeding, i.e., if the child was receiving only breast milk and no other liquid or solid food, including water and water-based drinks [28], on the day of the examination (yes vs. no), and mother's report of presence of at least one of the following breastfeeding problems in the first month postpartum: breast engorgement, pain while breastfeeding, cracked nipples, low or excessive milk supply, mastitis and baby’s suction difficulties (yes vs. no). Finally, in order to test the association between satisfaction with breastfeeding and PPD symptoms, multivariate Poisson regression analysis with robust variance was performed including all covariables showing association with maternal satisfaction with breastfeeding at p<0.1; adjusted prevalence ratios and their respective 95%CI were estimated. The cutoff point considered for the variable satisfaction with breastfeeding was the median score obtained in the whole sample: women scoring above the median were considered to have increased satisfaction.

The project was approved by the research ethics committees of the two hospitals involved in the study, namely Hospital de Clínicas de Porto Alegre (protocol 1288088) and Hospital Moinhos de Vento (protocol 1204288), both located in Porto Alegre, southern Brazil. All patients signed an informed consent form before their inclusion in the study.

## Results

Of the 354 puerperal women initially included in the study, 287 were interviewed at their homes the week after the child completed 30 days of life– 194 from the public and 93 from the private maternity. A total of 67 women were lost to follow-up, i.e., they were not reached (to arrange the interview) after three attempts via telephone contact on different days and times, in addition to one attempt in person at the address provided. These 67 women were similar to the ones interviewed with regard to type of delivery, parity, and newborn sex, but they had fewer years of schooling (p<0.01) and predominantly white skin color (p = 0.032) when compared to the women effectively included in the sample.

Table 1 shows the sociodemographic, gestational, and perinatal characteristics of the women who participated in the study. Maternal age ranged from 16 to 45 years, with a mean of 29 years; most mothers had white skin color, had completed at least secondary school, and lived with their husbands/partners. No predominance was observed in terms of socioeconomic classification, parity, or type of delivery.

**Table 1:** Sociodemographic, gestational, and perinatal characteristics.

Variable	n	%
Mother		
Age (years)	23	8.1
≤ 19		
20–34	199	69.3
≥ 35	65	22.6
Skin color		
White	216	75.3
Non-white	71	24.7
Socioeconomic level*		
A/B	163	56.8
C/D/E	122	42.8
Schooling^†^		
Primary school incomplete	31	10.8
Primary school complete	52	18.1
Secondary school complete	104	36.2
Higher education complete	100	34.8
Cohabitation with infant’s father		
Yes	248	86.4
No	39	13.6
Parity		
Primiparous	142	49.5
Multiparous	145	50.5
Planned pregnancy		
Yes	154	53.7
No	133	46.3
Type of delivery		
Vaginal	149	51.9
Cesarean	138	48.1
Newborn		
Sex		
Male	136	47.4%
Female	151	52.6%
Weight (grams), mean ± standard deviation	3,327 ± 433.84	

Distribution of sociodemographic, gestational, and perinatal characteristics, Porto Alegre, Brazil, 2016 (n = 287).

* Socioeconomic level was categorized according to Associação Brasileira de Empresas de Pesquisa [23]. Levels A and B indicate higher socioeconomic strata. Data missing for 2 participants.

^†^ Data missing for 2 participants.

The prevalence of exclusive breastfeeding at 30 days was 61.7%. Most women (59.2%) did not have difficulties to breastfeed. The prevalence of a positive screening test result for PPD was 12.5%.

MBFES scores ranged from 63 to 145, with a median of 124. The following covariables showed association with increased maternal satisfaction (i.e., scored above the median) at p<0.1: lower maternal age, non-white maternal skin color, cohabitation with the infant’s father, planned pregnancy, vaginal delivery, exclusive breastfeeding at 30 days, absence of breastfeeding problems, and a negative screening test result for PPD (Table 2). Type of hospital (public or private) was the only covariable not associated with the outcome.

**Table 2:** Association between maternal satisfaction with breastfeeding and postpartum depression.

Variables	Total sample, n (%)	Satisfaction with breastfeeding,* n (%)		Crude PR (95%CI)	p	Adjusted PR^†^ (95%CI)	p
		Lower	Higher				
Maternal age	287 (100)			0.99 (0.97–1.00)	0.099	0.98 (0.97–1.00)	0.062
Maternal skin color					0.014		0.087
White	216 (75.3)	100 (46.3)	116 (53.7)	1		1	
Non-white	71 (24.7)	22 (31.0)	49 (69.0)	1.29 (1.05–1.57)		1.19 (0.98–1.45)	
Cohabitation with infant’s father					0.030		0.095
Yes	248 (86.4)	98 (39.5)	150 (60.5)	1.57 (1.04–2.37)		1.42 (0.94–2.14)	
No	39 (13.6)	24 (61.5)	15 (38.5)	1		1	
Planned pregnancy					0.079		0.110
Yes	154 (53.7)	58 (37.7)	96 (62.3)	1.20 (0.98–1.47)		1.18 (0.96–1.44)	
No	133 (46.3)	64 (48.1)	69 (51.9)	1		1	
Type of delivery					0.083		0.319
Vaginal	149 (51.9)	56 (37.6)	93 (62.4)	1.19 (0.98–1.47)		1.12 (0.89–1.39)	
Cesarean	138 (48.1)	66 (47.8)	72 (52.2)	1		1	
Exclusive breastfeeding at 30 days					<0.001		0.001
Yes	177 (61.7)	56 (31.6)	121 (68.4)	1.71 (1.33–2.19)		1.52 (1.18–1.95)	
No	110 (38.3)	66 (60.0)	44 (40.0)	1		1	
Breastfeeding problems					0.001		0.031
Yes	117 (40.8)	65 (55.6)	52 (44.4)	1		1	
No	170 (59.2)	57 (33.5)	133 (66.5)	1.49 (1.19–1.88)		1.28 (1.02–1.59)	
Postpartum depression screening					0.043		0.047
Positive	36 (12.5)	22 (61.1)	14 (38.9)	1		1	
Negative	251 (87.5)	100 (39.8)	151 (60.2)	1.55 (1.02–2.36)		1.47 (1.01–2.16)	

95%CI = 95% confidence interval; PR = prevalence ratio.

Association between maternal satisfaction with breastfeeding and postpartum depression screening test results: crude and adjusted prevalence ratios, Porto Alegre, Brazil, 2016.

* The cutoff point was the median score obtained in the sample: ≥ 124.

^†^ All covariables showing association with maternal satisfaction with breastfeeding at p<0.1 were included in the model.

The multivariate analysis revealed an association between maternal satisfaction with breastfeeding and PPD symptoms. The prevalence of increased satisfaction with breastfeeding was 47% higher among women who screened negative for PPD when compared to those who screened positive for the disorder (adjusted prevalence ratio 1.47; 95%CI 1.01–2.16; Table 2).

## Discussion

Confirming our hypothesis, an association was found between increased maternal satisfaction with breastfeeding and a negative screening test result for PPD symptoms. However, this being a cross-sectional study, it was not possible to infer causality.

We believe that a lower level of maternal satisfaction with the breastfeeding process may cause or aggravate depressive symptoms. Satisfaction with breastfeeding is a measure of the woman’s own perception of her breastfeeding experience. Mothers often have the expectation of an easy, uneventful, pleasurable breastfeeding experience. Therefore, when faced with difficulties, these mothers tend to feel guilty, frustrated, and sad, perceiving those difficulties as failure. These feelings, in turn, may increase the risk for PPD [29], as observed by Pope and Mazmanian [18] in a study conducted in Canada.

Maternal satisfaction with breastfeeding is important both for the maintenance of this practice [5] and for its success [30]. In the study conducted by Cooke et al. [5], the authors considered maternal perception as a critical point for both satisfaction with breastfeeding and the decision to wean. According to those authors, breastfeeding problems alone did not show association with weaning, but women with lower levels of satisfaction showed a 3- to 15-fold higher chance of interrupting breastfeeding [5].

According to some authors, breastfeeding success from a maternal point of view is more related to maternal satisfaction with breastfeeding than to breastfeeding duration or exclusivity [4, 6, 30]. Nevertheless, studies exploring maternal satisfaction with breastfeeding are scarce. Some studies have been able to identify factors associated with maternal satisfaction, e.g., a successful previous experience with breastfeeding, maternal perception of the baby’s progress, ability to deal with breastfeeding difficulties, autonomy and support to their decision on how to breastfeed, not feeling pressured by the community or by health professionals, self-confidence in their ability to breastfeed, in addition to prepartum intention to breastfeed [4, 30, 31]. In Japan, intra-hospital practices of skin-to-skin contact, rooming in, and encouragement to breastfeed on demand were found to increase maternal satisfaction with breastfeeding [6]. According to the authors, increased levels of maternal satisfaction with breastfeeding could be related to the improved maternal-infant bonding promoted by these practices.

Notwithstanding, reverse causality cannot be ruled out. PPD symptoms may magnify the mother’s perception of breastfeeding difficulties and also compromise the interaction between mothers and their children [29], thus leading to lower levels of satisfaction. Some evidence has been published, especially over the last 30 years, about the association between PPD symptoms and early weaning. In all 48 manuscripts included in a meta-analysis on the topic [14], an association was found between early interruption of breastfeeding (both exclusive and non-exclusive) and PPD. Another important aspect addressed in that meta-analysis was the association between short breastfeeding duration, depression during pregnancy, and PPD. In that study’s conclusion, the authors state that women who present depressive symptoms before or after childbirth are at high risk to discontinue breastfeeding [14]. It is possible that maternal satisfaction with breastfeeding be involved in the association between early weaning and PPD symptoms. However, no study has so far explored the possibility of an association between maternal satisfaction with breastfeeding and PPD symptoms. To the authors’ knowledge, this is the first study to explore this association.

Some limitations of this study should be noted. First, because this was a cross-sectional study, the findings do not allow to identify the direction of the association observed. We believe in the existence of a two-way relationship between maternal satisfaction with breastfeeding and depressive symptoms, leading to a vicious cycle that may culminate in early weaning. Another limitation of the study was the exclusion of women who resided in dangerous/violent areas, which may have affected the external validity of the findings. Therefore, and also because of the participants lost to follow-up, caution is necessary when generalizing the present results to other populations. Also, this study did not investigate the causes of maternal dissatisfaction with breastfeeding and PPD. It is possible that the association found is modulated by the different causes involved. Studies with appropriate designs are necessary to elucidate the factors involved in the association.

## Conclusions

The association found in this study between maternal satisfaction with breastfeeding and PPD symptoms reinforces the importance of assessing the mental health of lactating mothers and their level of satisfaction with breastfeeding. In this scenario, one important step is to identify, already during pregnancy or during breastfeeding, women at a higher risk of developing PPD. In this sense, collecting data on the mother’s psychosocial environment, including information on her support network, and the mental health of her husband/partner, is extremely important and necessary.

Further studies are warranted, with different designs and different population profiles, to improve our knowledge of the association found in the present study and clarify its possible relationship with breastfeeding duration.

## Supporting information

S1 File (XLSX)
